# Supplementary material for: Adherence to Dietary Recommendations after One Year of Intervention in Breast Cancer Women: The DIANA-5 Trial
Source: Nutrients. 2021 Aug 27;13(9):2990. doi: 10.3390/nu13092990 (PMC8468802; doi:10.3390/nu13092990)
Supplement: Supplementary file 1 [file nutrients-13-02990-s001.zip › nutrients-1338302-supplementary.pdf]

EXAMPLES OF DIANA-5 LUNCH

WINTER MENU

Miso soup with onions

Brown rice with pumpkins cream and leeks

Mackerel patè

Durum wheat bread croutons

Marinated vegetables with humeboshi vinegar

Stewed vegetales

Cereal coffee

SUMMER MENU

Fresh salad

Basmati rice with vegetables

Chickpeas humus

Marinated vegetables with humeboshi vinegar

Stewed vegetales

Fruit kanten

	Winter Menu	Summer Menu	*LARN
**Kcal**	496	525	
**Protein**	16%	13%	12-18%
**Total fat**	29%	28%	25-30%
**Saturated**	2%	4%	10 %
**Monounsaturated**	13%	2%	
**Polyunsaturated**	5%	5%	5-10%
**Carbohydrate**	55%	59%	45-60%
**of which sugars**	12%	13%	< 15%
**Fiber**	12.5 g	9.0 g	30 g
**Cholesterol**	20.8 mg	0 mg	< 300 mg

*LARN – about 30% of daily intake of 1870kcal (Levels of reference intake of nutrients and energy for women age 30-74 years)
